# Health Seeking Behaviour and Associated Factors among Pulmonary Tuberculosis Suspects in Lay Armachiho District, Northwest Ethiopia: A Community-Based Study

**DOI:** 10.1155/2016/7892701

**Published:** 2016-02-28

**Authors:** Eshetu Haileselassie Engeda, Berihun Assefa Dachew, Hiwot Kassa Woreta, Mengistu Mekonnen Kelkay, Tesfaye Demeke Ashenafie

**Affiliations:** ^1^Department of Nursing, College of Medicine and Health Sciences, University of Gondar, P.O. Box 196, Gondar, Ethiopia; ^2^Department of Epidemiology and Biostatistics, Institute of Public Health, College of Medicine and Health Sciences, University of Gondar, P.O. Box 196, Gondar, Ethiopia

## Abstract

Studies in the northern part of Ethiopia showed high prevalence of undiagnosed cluster of tuberculosis cases within the community which demanded an investigation of the health care seeking behaviour of tuberculosis suspects. A community-based cross-sectional study was conducted in Lay Armachiho district, Northwest Ethiopia. Individuals who had cough for at least two weeks and aged greater than or equal to 15 years were included in the study. Data were collected by interview using pretested and structured questionnaire. Logistic regression was computed and adjusted odds ratio with 95% confidence interval was calculated. Out of the total population surveyed (29, 735), 663 (2.2%) individuals were found to be pulmonary tuberculosis suspects. Majority of the suspects reported that they had visited a modern health care facility. Those aged 15 to 34 and aged 35–54 had secondary educational level and above; those who were civil servants, those who were farmers, those who had previous history of tuberculosis treatment, and those who perceived that they were sick were more likely to visit a modern health care facility. The proportion of respondents who had taken traditional measures was found to be higher than some other districts. Improving the socioeconomic status of the community is recommended.

## 1. Background 

According to the 2015 World Health Organization (WHO) global tuberculosis (TB) report, TB remains a major global health problem that killed 1.5 million people in 2014 alone. This report also showed that an estimated number of 9.6 million new tuberculosis cases are diagnosed in the same year [1].

Evidences showed that over 81% of TB related morbidity and mortality occurred in developing countries [2]. This enormous burden of TB was mainly because of high prevalence of human immune deficiency virus infection [3], emergence of multidrug resistant (MDR) TB, social inequalities, ineffective TB control efforts, and low TB case detection rates [2, 4]. Moreover, many studies indicated that reservoirs for the extensive transmission of TB found predominantly in the community are being undiagnosed [2, 4, 5].

In Ethiopia, TB is the major cause of hospital admission and mortality [6]. The country stands 7th among the 22 high TB burden countries and grouped among the 27 high MDR tuberculosis burden countries [1]. The estimated annual incidence and prevalence of TB in Ethiopia is 258 and 237 per 100,000 population, respectively [6]. A TB trend analysis study also indicated that the incidence rate of TB in Ethiopia was increasing at a rate of 5 new TB cases per 100,000 population per year [7].

Ethiopia adopted the internationally recommended TB control strategy, the Directly Observed Treatment Short (DOTS) course, by early 1900s. The major components of this strategy included case detection by sputum smear microscopy and standardized treatment with supervision [6]. However, despite the early introduction of such an important strategy, a recent national report showed a TB case detection rate of 58.9% which is below the national target of detecting 82.7% of infectious TB cases [8]. Low TB case detection rate may be related to individual or organizational factors. That is to say, either the patients fail to seek health care or the health care system fails to address TB case detection particularly at the community level [9]. In one or another way, this usually ends up with a delay in diagnosis of TB patients.

Health seeking behaviour is defined as any action taken by the individual TB suspect to get relief from his/her presenting symptoms [10]. This action may include any form of traditional illness management including self-treatments by taking or applying local remedies, visit to traditional healers, “Holy Water,” local injectors, and/or buying medications from drug stores without prescription. On the other hand, people with TB symptoms may visit government or private modern health care facilities to get prescribed treatment [9, 10].

The health care seeking behaviour of TB suspects varies from country to country. In Ukraine, for instance, 88% of the respondents reported that they visited polyclinic or hospital as a primary choice [11]. In Northern India, 87% of the study participants reported that they had taken some kind of self-initiated action such as home remedies [10]. In another Indian study, 72% of respondents reported that they had consulted nonprofessional private healer [12]. In Vietnam, TB case suspects visits to pharmacy and private practitioner were the initial actions for alleviating TB symptoms [5]. In Gambia, majority of people from the study population seek help from modern health institutions early [13]. Respondents from Uganda reported that they used self-treatment as a primary choice and visited health institutions when they became bedridden in most cases [14]. A study done in Ethiopia demonstrated that 60% of the respondents visited modern health institutions while 22% and 18% of the respondents sought traditional healers and took no action, respectively [9].

There are several factors which can affect the health seeking behaviour of TB suspects. According to the literature, health seeking behaviour is a dynamic process which is influenced by cultural, religious, sociodemographic, environmental, political, and other issues [9]. These factors shape the behaviour of individual patients by influencing their perception about symptoms. Moreover, their decision on the choice of a particular health care action is largely influenced by those factors [15].

In relation to the health seeking behaviour of TB suspects, a considerable degree of diagnosis and treatment delay had been observed in some studies. For instance, the median time between the start of symptoms and first visit to health care provider in Ukraine and Ethiopian studies was 30 days [9, 11]. A delay in visiting modern health care facilities results in a delay in detecting infectious tuberculosis [16]. This in turn results in a more complicated disease process, increased patient suffering, extensive transmission of the infection within the community, and higher risk of TB associated mortality [17].

Various strategies had been tried in the past to bring the health seeking behaviour of TB suspects on the right track and to overcome diagnostic delays. Among these, public health education, identifying target groups for health education, and targeting vulnerable and difficult to reach groups were the major ones [5, 10, 11].

In Ethiopia a significant number of facility-based studies had been conducted on the health seeking behaviour of newly diagnosed TB patients. However, community-based studies are rarely available at national level in general and no information is found at the current study area in particular. Nevertheless, the health seeking behaviour of TB suspects who preferred to stay within the community may be different as compared to those who visited modern health care facilities. Therefore, this study will be immensely important to describe the level of modern health care seeking practice in the rural community and factors associated with it.

## 2. Materials and Methods 

A community-based cross-sectional study was conducted from January 17 to February 24, 2014, in the rural kebeles (the lowest administrative units) of Lay Armachiho district. The district is found in North Gondar administrative zone, Amhara Regional State, 768 km far from Addis Ababa to the Northwest of Ethiopia. The total population of the district was estimated to be 175,114. Within the district there are 33 rural kebeles and one urban kebele. With regard to health facilities, there were 34 health posts (one per each kebele) and 7 health centres. According to the health care delivery system of Ethiopia, health posts are the lowest units and do not have sputum diagnostic facility for tuberculosis. On the other hand a health centre, serving as a referral centre for five satellite health posts in its catchment area, is supposed to have a sputum diagnostic facility for pulmonary tuberculosis. However, only two out of the seven health centres had sputum diagnostic facility during the study period.

All individuals aged above 15 who had experienced cough for two or more weeks during the time of the survey were included in the study.

The outcome variable of this study is “modern heath care facility visit.” The independent variables include sociodemographic characteristics (age, sex, religion, marital status, occupation, and educational level) and illness related factors (history of hemoptysis, previous history of TB treatment, and perceived health status).

Sample size was determined by using single population proportion formula by considering the following assumptions: 95% confidence interval (CI), 0.22% proportion (prevalence of TB in rural Northern Ethiopia [18]), 0.1% margin of error, a design effect of 2, and a 10% nonresponse rate, and the minimum sample size was 18,553 individuals.

Cluster sampling technique was utilized in this study. From the 33 rural kebeles (clusters) under the administration of Lay Armachiho district, 9 kebeles (27.3%) were selected by simple random sampling. Then all individuals (within the target age group) found within the randomly selected nine kebeles were surveyed house to house.

Data were collected by pretested and structured Amharic version questionnaire (Amharic is the local language of the district spoken by all people in the study kebeles) using interview technique. Eighteen health extension workers and nine BSc nurses were recruited for data collection and supervision, respectively. Before data collection, two data collectors were assigned to each kebele. Then each data collector conducted a house-to-house survey among the randomly selected households. During the survey, the head of the household was asked about whether or not any family member had cough for a duration more than or equal to two weeks. When such a case was found, the data collectors interviewed suspects using the questionnaire about TB symptoms, sociodemographic variables, and the different health care actions taken. Repeat visits were conducted for those individuals who were not available during the first visit. At each data collection spot, sufficient explanation about the aim of the research was given to the study participants before the interview.

The quality of data was assured by applying the following methods. First, the questionnaires were carefully designed based on the literature. Second, pretest was conducted at one of the rural kebeles of the nearby district equivalent to 5% of the sample size. Third, appropriate modifications were made on the questionnaires after viewing the pretest result. Fourth, a two-day training was given for data collectors and supervisors just before the actual data collection time. Every day after data collection, questionnaires were reviewed and checked for completeness, accuracy, and clarity by the supervisors and principal investigator and the necessary feedback was offered to data collectors in the next morning.

The collected data were coded, entered into Epi Info 7, and exported to SPSS version 20 software for analysis. Descriptive statistics was used to illustrate the means, standard deviations, medians, and frequencies of the study variables. Bivariate analysis was computed and those variables whose *P* values were less than or equal to 0.2 were fitted into backward multivariate logistic regression model. Odds ratios with 95% confidence interval were used to determine the strength of association between dependent and independent variables. *P* values less than or equal to 0.05 were considered statistically significant.

In this study, when respondents reported that they visited a health post, a health centre, a private clinic, or a hospital, it had been taken as they visited a modern health care facility. On the contrary, when they reported that they had taken actions such as self-treatment with home remedies, bought medicines without prescription, took traditional medicine (herbs), went to “Holy Water,” and took no action, it was considered as if they had taken traditional measures.

Ethical clearance was obtained from institutional ethical review board of College of Medicine and Health Sciences, University of Gondar. Moreover, letter of support was secured from the woreda health office and each kebele administration. In addition to that, following an explanation of the purpose, the benefits, and the possible risks of the study, consent was obtained from all study subjects who assured that participation was on voluntarily basis. On top of that, to keep the anonymity of study participants, code numbers rather than personal identifiers were used and all questionnaires were sealed with post following data collection at each kebele every day. When TB suspects were identified during the survey, they were counseled in order to go to the nearby health centre (for those health centres which have sputum examination facility) or hospital for sputum examination. Finally, the questionnaires were kept locked all the time except during data entry.

## 3. Results

### 3.1. Sample Characteristics and Prevalence of PTBC Suspects

A total of 29,735 illegible individuals participated in the study of whom the majority were male, 15,833 (53.2%). Out of those study participants, 663 (2.2%) individuals were fund to be pulmonary tuberculosis suspects. The prevalence of PTBC suspects among male and female participants was 1.3 and 3.3, respectively. The median age (IQR) of PTBC suspects was 37 (32–45 years). Majority of PTBC suspects were females (68.8%), married (71.9%), and Orthodox Christian followers (74.1%). Approximately 58% of PTBC suspects attended elementary school education and 48% of them were unemployed (Table 1).

### 3.2. Duration of Cough and Past History of TB Treatment

In this study, the median duration of cough (IQR) was 3 weeks (2–4 weeks). Four hundred ninety-nine (75.3%), 125 (18.9%), 17 (2.6%), and 22 (3.3%) of PTBC suspects had a cough that lasted 1–4 weeks, 2–5 weeks, 9–12 weeks, and greater than 12 weeks, respectively. One hundred fifty-two (22.9%) of the PTBC suspects had previous history of TB treatment.

### 3.3. Perception of Illness

Among the total PTBC suspects who had been asked about perception of their symptoms, 177 (26.7%) perceived their symptom as common cold. One hundred sixty-four (24.7%) reported that their symptoms were due to “bird” or “Nefas,” a common local description of an illness believed to be resulting from exposure to cold weather or wind. Similarly, 130 (19.6%) of the PTBC suspects reported that their symptoms were due to “mitch” or “gerefta,” another local description of illness given to an illness similar to pneumonia (usually treated traditionally by hot steam in which leaves will be boiled in water and the patient will inhale the steam). One hundred thirty-nine (21%), 30 (4.5%), and 15 (2.3%) of the respondents perceived that their symptoms were because of pneumonia, asthma, and pulmonary tuberculosis, respectively, whereas 8 (1.2%) reported that they had no disease.

### 3.4. Health Care Seeking Behaviour

All respondents were asked about the first action they had taken to alleviate their symptoms. The majority of PTBC suspects (60.9%) visited modern health care facilities first whereas the rest of PTBC suspects took some other kind of individual action which included but not limited to home treatment and traditional medicine (Table 2). The median time (IQR) elapsed to take first action after the onset of symptoms was 13 days (10–17).

### 3.5. Bivariate and Multivariate Findings: Factors Associated with Health Care Seeking Behaviour

In the bivariate analysis, age (those younger than 55 years), occupation (farmers and civil servants), having history of hemoptysis, perceived health status (perceived wellness), and having previous history of TB treatment showed statistical significance with visiting modern health care facility. However, in the multivariate model, age (15–34 years and 35–54 years), educational level (secondary and above), occupation (farmers and civil servants), perceived health status (perceived wellness), and having previous history of TB treatment are yielded as factors associated with visiting modern health care facility among PTBC suspects.

Age was significantly associated with visiting modern health care facility. Pulmonary tuberculosis suspects aged 15 to 34 and aged 35–54 were more likely to visit a modern health care facility than PTBC suspects aged 55 and above (AOR (95% CI) 4.59 (2.71–7.76) and 2.45 (1.54–3.88), resp.). The other significant factor was level of education with respondents who had secondary educational level and above and were more likely to visit a modern health care facility than those who were illiterate (AOR (95% CI) 2.61 (1.35–5.04)). Occupation was also among the significant factors as PTBC suspects who were civil servants or farmers were more likely to report that they visited a modern health care facility than PTBC suspects who were unemployed (AOR (95% CI) 3.02 (1.26–5.58) and 1.43 (1.01–2.05), resp.). History of TB treatment was also significantly associated with visiting a modern health care facility. Pulmonary tuberculosis suspects who had previous history of TB treatment were more likely to visit a modern health care facility than PTBC suspects who had no previous history of TB treatment (AOR (95% CI) 3.47 (2.16–5.58)). Perceived health status was also among the significant factors as PTBC suspects who perceived they were sick were more likely to visit a modern health care facility than who perceived they were well (AOR (95% CI) 3.19 (1.98–5.13)) (Table 3).

## 4. Discussion

In developing countries like Ethiopia where TB case detection rate is below the WHO set value and majority of the population live in rural areas in which TB diagnostic facilities are not fulfilled, understanding the health care seeking behaviour of PTBC suspects could be very crucial in order to design appropriate behaviour modification mechanisms.

In this study 39.1% of PTBC suspects had not visited modern health care facilities during the study period. This might imply that the respondents either did not consider their symptoms as a serious health problem or they had used other traditional methods to alleviate their symptoms. Although this finding is lower than a West Gojjam (Northwest Ethiopia) study [9], it is higher than a study done in Dabat district (Northwest Ethiopia) [19] in which the proportions of PTBC suspects who had not visited modern health care facility were 40% and 20%, respectively. Despite a relatively similar geographic location, significant variations have been observed between the Dabat and the current studies. One possible explanation for these discrepancies might be due to the fact that strategies to increase community awareness about tuberculosis might not be implemented uniformly and effectively across districts. On the other hand, a variation in socioeconomic and sociocultural variables which affect medical health care seeking behaviour among districts could partly explain the situation.

In our study, more than 50% of PTBC suspects described their symptoms as “bird” or “gerefta.” This finding is in line with another Ethiopian study in that 46% of respondents reported their symptoms as “bird” [9]. This implies that knowledge about TB symptoms and when to visit a medical care provider is low among the majority of the population in the study area. As acknowledged by other studies [6, 19, 20], this in turn will lead to misconception about TB within the community, delay in modern health care seeking, and as a result increased period of communicability.

In this study, PTBC suspects aged 55 years and older were less likely to visit a modern health care facility than those who were younger than 55 years. In most parts of Ethiopia in general and in the Northern part of the country in particular, it is not uncommon to see traditional treatment of illness among the elderly community by either traditional healers or “Holy Water.” On the other hand, unlike the younger age groups, the elderly people hardly get access to information about TB symptoms through modern education and peer education and, therefore, misconceptions are common in this age group. Literature also showed a downward trend of knowledge about health related matters as age increases [21]. Moreover, the fact that elderly people are economically dependent on others and may refrain from visiting modern health care facilities could also be one possible explanation for the finding.

Consistent with other local and international studies [22, 23], the current study also showed that respondents who had secondary educational level and above were more likely to visit modern health care facility than those who were illiterate. This is due to the fact that people at this educational level usually have higher chance of accessing health related information easily from various media and through their formal education. Moreover, the more the educational level of people is, the better their understanding of diseases processes, availability of diagnosis, treatment options, and the risk of delay in medical care seeking will be [21].

In this study, those who had some kind of occupation (civil servants or farmers) were more likely to visit a modern health care facility than those who were unemployed. The possible explanation for this finding might be attributed to the fact that when people have some kind of job which generates income, they could be motivated to visit a modern health care facility. This is usually because of direct (e.g., cost of medicine) and indirect (e.g., transport) costs associated with modern health care facility. Moreover, apart from relatively affording medical health care service associated costs, those people who had job and were able to generate better income may have television or radio or both to access appropriate information about TB [19].

In line with another Ethiopian community-based survey [9], our study also showed that PTBC suspects who had previous history of TB treatment were more likely to visit a modern health care facility than PTBC suspects who had no previous history of TB treatment. One possible reason for this finding could be the fact that preexisting knowledge regarding the seriousness of TB infection, its causative agent, and the availability of services (e.g., free of charge diagnosis and treatment in Ethiopian context) shaped their health seeking behaviour and influenced their decision to visit a modern health care facility [24].

In this study, respondents who had perceived that they were sick were more likely to visit a modern health care facility than those who perceived that they were well. The possible explanation for this finding might be that the high risk perception of PTBC suspects could ultimately lead them to visit a modern health care facility as supported by a study conducted in Bangladesh [24].

One of the possible limitations of this study could be its cross-sectional nature in which it does not confirm definitive cause and effect relationship. Moreover, since the major of the study participants had no formal (modern) education and we used self-reported data, the reliability and validity of the results might be influenced to some extent.

## 5. Conclusion

This study revealed that the majority of pulmonary tuberculosis suspects had visited modern health care facility; however, the proportion of respondents who had taken traditional measures was still higher as compared with some other districts. Younger age, educational level (secondary and above), occupation (farmers and civil servants), perceived health status (perceived wellness), and having previous history of TB treatment were factors significantly associated with visiting modern health care facilities. Interventions aimed at reducing unemployment rate, increasing literacy rate, and improving the socioeconomic status of communities in the district are recommended for the sustainable modification of health care seeking behaviour of TB suspects.

## Figures and Tables

**Table 1:** Sociodemographic characteristics of pulmonary tuberculosis suspects in Lay Armachiho district, Northwest Ethiopia, 2014.

Characteristics	Number	Percent (*n* = 663)
Age		
15–34	183	27.6
35–54	375	53.8
55 and above	123	18.6
Sex		
Male	207	31.2
Female	456	68.8
Marital status		
Unmarried^a^	186	28.1
Married	477	71.9
Religion		
Orthodox Christian	491	74.1
Muslim	107	16.1
Protestant	65	9.8
Educational level		
Illiterate	193	29.1
Elementary	382	57.6
Secondary and above	88	13.3
Occupation		
Unemployed^b^	318	48
Farmer	300	45.2
Civil servant	45	6.8

^a^Unmarried includes single (never married), widowed, separated, and divorced.

^b^Unemployed includes those who have no job and students.

**Table 2:** First action taken by pulmonary tuberculosis suspects in Lay Armachiho district, Northwest Ethiopia, 2014 (*n* = 663).

Action taken	Number	Percent
Visited health post	152	22.9
Visited health centre	190	28.7
Visited hospital	16	2.4
Visited private clinic	46	6.9
Self-treatment with safe home remedies (tea, honey, etc.)	53	8
Bought drugs without prescription	7	1.1
Taken traditional medicine (herbs)	70	10.6
Went to “Holy Water”	92	13.9
No action taken	37	5.6
Total	663	100

**Table 3:** Bivariate and multivariate logistic regression analysis of factors associated with visiting modern health care facility among pulmonary tuberculosis suspects in Lay Armachiho district, Northwest Ethiopia, 2014.

Variable	Visited modern health care facility first		Crude odds ratio with 95% CI	Adjusted odds ratio with 95% CI
	Yes	No		
Age				
15–34	135	48	4.87 (2.97–7.98)	4.59 (2.71–7.76)^*∗∗∗*^
35–54	224	133	2.91 (1.90–4.46)	2.45 (1.54–3.88)^*∗∗∗*^
55 and above	45	78	1.00	
Sex				
Male	134	73	1.00	
Female	270	186	0.791 (0.56–1.11)	1.14 (0.72–1.80)
Marital status				
Unmarried^a^	120	66	1.00	
Married	284	193	0.80 (0.56–1.15)	0.84 (0.57–1.26)
Religion				
Orthodox Christian	290	201	1.00	
Muslim	74	33	1.55 (0.99–2.43)	0.76 (0.41–1.14)
Protestant	40	25	1.10 (0.65–1.88)	0.98 (0.46–2.08)
Educational level				
Illiterate	125	68	1.00	1.00
Elementary	219	163	0.73 (0.5–1.04)	1.10 (0.72–1.67)
Secondary and above	60	28	1.16 (0.68–1.99)^a^	2.61 (1.35–5.04)^*∗∗*^
Occupation				
Unemployed^b^	172	146	1.00	1.00
Farmer	195	105	1.57 (1.14–2.17)	1.43 (1.004–2.05)^*∗*^
Civil servant	37	8	3.92 (1.7–8.69)	3.02 (1.26–7.25)^*∗*^
History of TB treatment				
Yes	120	32	2.99 (1.95–4.59)	3.47 (2.16–5.58)^*∗∗∗*^
No	284	227	1.00	1.00
Hemoptysis				
Yes	28	8	2.33 (1.04–5.20)	2.57 (0.99–6.63)
No	376	251	1.00	
Perceived health status				
Well	50	67	1.00	1.00
Sick	354	192	2.47 (1.4–3.70)	3.16 (1.98–5.13)^*∗∗∗*^

^a*∗*^ *P* value < 0.05; ^*∗∗*^ *P* value < 0.001; ^*∗∗∗*^ *P* value < 0.0001.

^b^Unemployed includes those who do not have any kind of job and students.
